# Tooth loss and cancer risk: a dose–response meta analysis of prospective cohort studies

**DOI:** 10.18632/oncotarget.23850

**Published:** 2017-12-16

**Authors:** Jun Shi, Weidong Leng, Lunhua Zhao, Cai Deng, Chenli Xu, Jue Wang, Yu Wang, Xingchun Peng

**Affiliations:** ^1^ Department of Stomatology, Taihe Hospital, Hubei University of Medicine, Shiyan, Hubei, 442000, China; ^2^ School of Basic Medical Sciences, Hubei University of Medicine, Shiyan, Hubei, 442000, China; ^3^ Department of Ultrasonography, Xiangyang No.1 People’s Hospital, Hubei University of Medicine, Xiangyang, Hubei, 441000, China; ^4^ Department of Oncology, Suizhou Hospital, Hubei University of Medicine, Suizhou, Hubei, 441300, China

**Keywords:** cancer, tooth loss, dose–response relationship, meta analysis

## Abstract

Conflicting results to identify the relationship between tooth loss and cancer risk. Therefore, a dose-response meta-analysis was performed to clarify and quantitative assessed the correlation between tooth loss and cancer risk. Up to March 2017, 25 observational epidemiological studies were included in current meta-analysis. Tooth loss was significantly associated with a higher risk of cancer. Additionally, tooth loss was associated with significantly a higher risk of esophageal cancer, gastric cancer, head and neck cancer, colorectal cancer, pancreas cancer, lung cancer, prostate cancer, bladder cancer and hematopoietic cancer. Subgroup analysis showed consistent findings. Furthermore, a significant dose-response relationship was observed between tooth loss and cancer risk. Increasing per 10 of tooth loss was associated with a 9% increment of cancer risk, 14% increment of esophageal cancer risk, 9% increment of gastric cancer risk, 31% increment of head and neck cancer risk, 4% increment of colorectal cancer risk, 7% increment of pancreas cancer risk, 19% increment of lung cancer risk, 2% increment of bladder cancer risk and 3% increment of hematopoietic cancer risk. Considering these promising results, tooth loss might be harmful for health. Large sample size, different ethnic population and different cancer type are warranted to validate this association.

## INTRODUCTION

Cancer has to be the second-leading cause in 2015 that caused over 8.8 million deaths worldwide in 2015 [1]. Due to countries geographical environment, living habits, cancer incidence is very different. The incidence of cancer in developing countries such as Africa, Asia and South America is the most severe. A total of 14 million cancer cases were added worldwide and 8.2 million people died in 2012. Among them, China added 3.07 million cancer patients and caused about 2.2 million deaths, accounting for 21.9% of the total global deaths. The incidence of cancer in developed countries is still higher than in developing countries. Residents of North America and Europe are the most vulnerable to cancer, but China has a large population base, making it the country with the highest number of cancer deaths throughout the world. As more and more people in developing countries improve their living standards and dietary patterns change, the chances of cancer have increased significantly in developing countries [2]. The etiology of cancer involves both genetic and environmental factors. Therefore, understanding the impact of environmental factors on cancer will help to prevent cancer.

Oral cavity is an important part of the body, and is starts in the digestive system, mainly by the lip and cheek, tongue and palate, salivary glands, teeth and jaw, with mastication, swallowing, speech and feeling, and other functions, which maintain the normal shape of maxillofacial. Oral health is an important part of human health. The World Health Organization (WHO) identifies dental health as one of the top ten criteria for human health. Poor oral health may increase systemic inflammation, resulting in a local overly aggressive immune response, and thus could have important implications for cancer development. Periodontal disease and tooth loss are two common oral health measures [3]. Tooth loss has been considered to impact quality of life [4], and been known to considerably influence food choice, diet, nutrition intake, and esthetics [5].

Previous studies have examined the correlation between tooth loss and cancer risk [6–30]. However, the result remains controversial. Additionally, no study to quantitative assessed tooth loss in relation to cancer risk. Thus, we performed this dose-response meta-analysis to clarify and quantitative assessed the correlation between tooth loss and cancer risk.

## MATERIALS AND METHODS

This meta-analysis was conducted according to the Meta-analysis Of Observational Studies in Epidemiology (MOOSE) checklist [31].

### Search strategy

We included eligible studies to investigate the relationship between tooth loss and cancer risk in general adult populations. To develop a flexible, non-linear, r meta-regression model, we required that an eligible study should have categorized into 3 or more levels.

PubMed and EMBASE were searched for studies that contained risk estimates for the outcomes of cancer and were published update to March 2017, with keywords including “dentition” [MeSH] OR ”tooth loss” [MeSH] OR “edentulous” [MeSH] OR “lost of tooth” [MeSH] AND “cancer” [MeSH] OR “tumor” [MeSH] OR “neoplasms” [MeSH]. We refer to the relevant original essays and commentary articles to determine further relevant research.

### Study selection

Two independent researchers investigate information the correlation between tooth loss and cancer risk: outcome was cancer. Moreover, we precluded non-human studies, reviews, meta-analyses, editorials and published letters.

### Data extraction

Use standardized data collection tables to extract data. Each eligible article information was extracted by two independent researchers. We extracted the following information: first author; publication year; age; country; sex; cases and participants; the categories of tooth loss; relative risk or odds ratio (OR). We collect the risk estimates with multivariable-adjusted [32]. According to the Newcastle-Ottawa scale, quality assessment was performed for non-randomized studies [33]. The disagreements were resolved through consensus by all the authors.

### Statistical analysis

We pooled relative risk estimates to measure the association between tooth loss and cancer; the hazard ratio were considered equivalent to the relative risk [34]. Results in different subgroups of tooth loss and cancer risk were treated as two separate reports.

Due to different definitions cut-off points in the included studies for categories, we performed a relative risk estimates by the method recommended by Greenland, Longnecker and Orsini and colleagues [35]. Dose of tooth loss used the median tooth loss. If the median tooth loss category was not available, the midpoint of the upper and lower boundaries was considered the dose of each category. In addition, using restricted cubic splines to evaluate the non-linear association between tooth loss and cancer risk, with three knots at the 10th, 50th, and 90th percentiles of the distribution. A flexible meta-regression based on restricted cubic spline (RCS) function was used to fit the potential non-linear trend, and generalized least-square method was used to estimate the parameters. This procedure treats tooth loss (continuous data) as an independent variable and logRR of diseases as a dependent variable, with both tails of the curve restricted to linear. A *P* value is calculated for linear or non-linear by testing the null hypothesis that the coefficient of the second spline is equal to zero [32].

The between-study heterogeneity was assessed by *Q*-statistic (signifcance level at *P* ≤ 0.10) and the I^2^-statistic. STATA software 14.0 (STATA Corp, College Station, TX, USA) was using in all analyses. *P* < 0.05 was considered signifcant for all tests.

## RESULTS

### Literature search results

**Figure 1:**
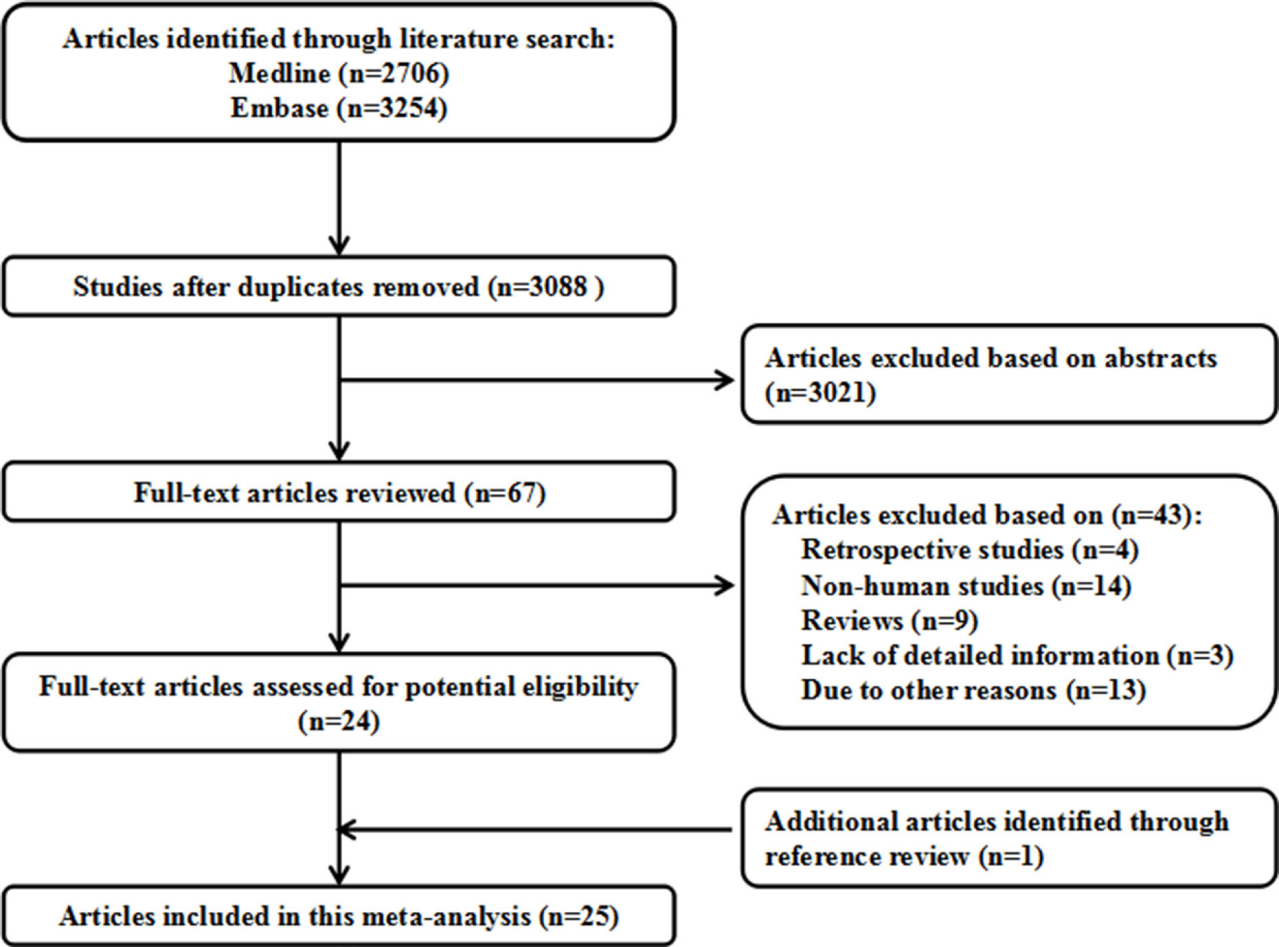
Flow diagram of the study selection process

We identifed 3088 relevant citations after removing duplicates. Reviewing their titles and abstracts, 3021 citations were excluded. The remaining 67 citations were assessed in more detail for eligibility by reading the full text. Among them, 43 were excluded, after review reference, one articles was included. Finally, 25 studies were used for the final data synthesis [6–30]. The flow chart of literature searching was presented in Figure 1, and the data were extracted. These studies were published update to March 2017.

### Study characteristics

The characteristics of the included studies of tooth loss and cancer risk are shown in the Table 1 and Supplementary Table 1. Among the selected studies, nine focused on esophageal cancer [6–13, 24], five focused on gastric cancer [6, 7, 12, 13, 15], eleven focused on head and neck cancer [11–13, 16–20, 23, 25, 26], four focused on colorectal cancer [12, 13, 28, 29], five focused on pancreas cancer [12–14, 21, 30], two focused on lung cancer [12, 13], two focused on prostate cancer [12, 13] and two focused on bladder cancer [12, 13]. Results in different subgroups were treated as two separate reports. Finally, Eighty-one independent reports from twenty five studies investigated the association between tooth loss and cancer.

**Table 1:** Characteristics of participants in included studies of tooth loss in relation to risk of cancer

Author (year)	Study design	Country	Sex of population	Age at baseline (years)	No of participants	Endpoints (cases)	Quality score
Abnet et al. (2001)	cohort	China	Mix	40–69	29548	Esophageal cancer (620) Gastric cancer (533)	8
Abnet et al. (2005)	cohort	Finnish	Mix	50–69	29124	Esophageal cancer (49) Gastric cancer (245)	8
Abnet et al. (2008)	case-control	Iran	Mix	> 18	843	Esophageal cancer (283)	6
Dar et al. (2013)	case-control	Kashmir	Mix	61.6	2367	Esophageal cancer (703)	6
Dye et al. (2007)	case-control	China	Mix	40–67	977500	Esophageal cancer (579)	6
Guha et al. (2007)	case-control	USA and Europe	Mix	any age	Europe (4110) USA (1852)	Europe: Esophageal cancer (91) Head and neck cancer (507) USA Esophageal cancer (95) Head and neck cancer (1457)	7
Hiraki et al. (2008)	case-control	Japan	Mix	58.0	15720	Head and neck (429) Esophagus (354) Stomach (702) Colon (662) Liver (167) Pancreas (178) Lung (909) Breast (756) Uterus (429) Ovary (103) Prostate (136) Bladder (62) Thyroid (121) Lymphoma (232)	7
Michaud et al. (2008)	cohort	USA	Male	40–75	48375	Total (5720) Lung (678) Oropharyngeal (118) Esophageal (131) Stomach (106) Pancreatic (253) Colorectal (1043) Kidney (271) Lung (678) Bladder (543) Prostate (541) Hematopoietic (934) Brain (132) Skin Melanoma (698) Non-Hodgkin lymphoma (524) Leukemia (250) Multiple myeloma (141)	8
Michaud et al. (2007)	cohort	USA	Male	40–75	51529	Pancreatic (216)	8
Shakeri et al. (2013)	case-control	Iran	Mix	40–75	922	Gastric (588)	6
Balaram et al. (2002)	case-control	India	Mix	22–58	1164	oral cavity (584)	5
Bundgaard et al. (1995)	case-control	Denmark	Mix	< 75	559	oral cavity (161)	6
Garrote et al. (2001)	case-control	Cuba	Mix	60	400	oral cavity (200)	5
Lissowska et al. (2003)	case-control	Poland	Mix	23–80	244	oral cancer (122)	6
Talamini et al. (2000)	case-control	Italian	Mix	27–86	274	oral cancer (131)	6
Stolzenberg-Solomon et al. (2003)	cohort	Finland	Male	50–69	29104	pancreatic cancer (174)	8
Bertrand et al. (2017)	cohort	USA	Male	40–75	51529	NonHodgkin lymphoma (875) Chronic lymphocytic leukemia/small lymphocytic lymphomas (290) diffuse large B-cell lymphomas (85) Follicular lymphomas (91)	8
Chen et al. (2016)	case-control	China	Female	20–80	1246	Oral cancer (250)	6
Chen et al. (2016)	case-control	China	Mix	40–85	1386	Esophagus (616)	6
Zuo et al. (2014)	case-control	China	Mix	> 18	317	Oral cancer (150)	7
Divaris et al. (2010)	case-control	USA	Mix	26–80	2650	Head and Neck Cancer (1361)	6
Liu et al. (2016)	case-control	China	Mix	20–74	5124	nasopharyngeal carcinoma (2528)	6
Momen-Heravi et al. (2017)	cohort	USA	Female	39–55	77443	colorectal cancer (1165)	8
Ren et al. (2016)	case-control	China	Mix	40–79	6619	colorectal cancer (1063)	6
Huang et al. (2016)	cohort	Sweden	Mix	20–70	19924	pancreatic cancer (126)	8

### Tooth loss and overall cancer risk

**Figure 2:**
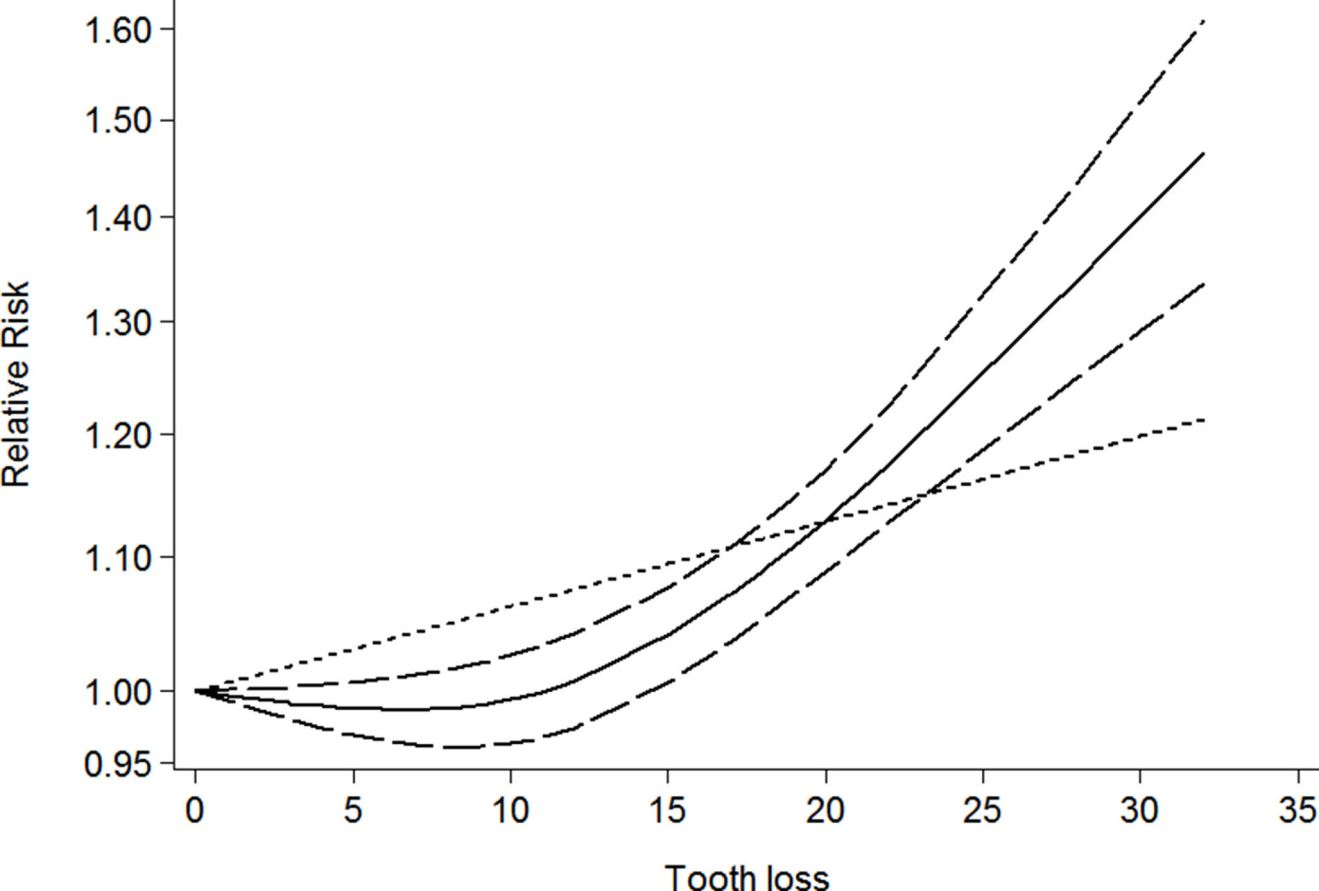
Dose-response relationship between tooth loss in relation to risk of overall cancer

Eighty-one independent reports from twenty five studies investigated the association between tooth loss and cancer [6–30]. Compared with the lowest tooth loss, tooth loss is significantly associated with a higher risk of cancer risk (RR:1.06; 95% CI, 1.02–1.09; *P* < 0.001) (Table 2). Additionally, a dose-response analysis revealed that each 10 tooth loss was associated with a 9% incremental in cancer risk (RR: 1.09; 95% CI, 1.05–1.13) (Figure 2). A cubic spline model revealed an positive non-linear correlation between tooth loss and cancer (*P* < 0.001 for non-linearity; Figure 2).

**Table 2:** Stratified analyses of relative risk of cancer

	No of reports	Relative risk (95% CI)	P for heterogeneity	I^2^	P for test
Total	81	1.06 (1.02–1.09)	0.000	51.2%	< 0.001
Subgroup analyses for cancer					
Esophageal cancer	10	1.18 (1.04–1.31)	0.558	0.0%	< 0.001
Subgroup analyses for Esophageal cancer					
Study location					
Caucasia	6	1.12 (1.02–1.21)	0.520	0.0%	< 0.001
Asia	4	1.26 (1.08–1.44)	0.363	5.9%	< 0.001
Study design					
Case–control	7	1.34 (1.04–1.64)	0.736	0.0%	< 0.001
Cohort	3	1.11 (1.04–1.18)	0.460	0.0%	< 0.001
Study quality					
Score ≥ 7	3	1.11 (1.04–1.18)	0.460	0.0%	< 0.001
Score < 7	7	1.34 (1.04–1.64)	0.736	0.0%	< 0.001
Gastric cancer	9	1.09 (1.03–1.16)	0.763	0.0%	< 0.001
Subgroup analyses for Gastric cancer					
Study location					
Caucasia	6	1.25 (1.11–1.36)	0.737	0.0%	< 0.001
Asia	3	1.04 (1.01–1.09)	0.863	0.0%	< 0.001
Study design					
Case–control	4	1.10 (1.02–1.19)	0.739	0.0%	< 0.001
Cohort	5	1.13 (1.04–1.23)	0.474	0.0%	< 0.001
Study quality					
Score ≥ 7	5	1.13 (1.04–1.23)	0.474	0.0%	< 0.001
Score < 7	4	1.10 (1.02–1.19)	0.739	0.0%	< 0.001
Head and neck cancer	19	1.52 (1.14–1.90)	0.000	71.3%	< 0.001
Subgroup analyses for Head and neck cancer					
Oral cancer	15	1.80 (1.30–2.30)	0.000	67.7%	< 0.001
Pharynx	2	1.14 (1.05–1.23)	0.295	5.7%	< 0.001
Larynx cancer	2	1.08 (1.02–1.15)	0.004	87.5%	< 0.001
Study location					
Caucasia	11	1.15 (1.04–1.26)	0.004	61.2%	< 0.001
Asia	8	1.82 (1.52–2.12)	0.288	18.6%	< 0.001
Study design
Case–control	18	1.52 (1.13–1.92)	0.000	72.7%	< 0.001
Cohort	1	1.60 (0.84–3.04)	0.288	18.6%	0.121
Study quality
Score ≥ 7	9	1.13 (1.04–1.23)	0.001	70.1%	< 0.001
Score < 7	10	1.91 (1.58–2.24)	0.171	29.8%	< 0.001
Colorectal cancer	13	1.07 (1.02–1.14)	0.114	33.5%	< 0.001
Subgroup analyses for Colorectal cancer					
Colon	5	1.09 (1.02–1.17)	0.330	13.2%	< 0.001
Rectal	3	1.08 (1.01–1.17)	0.082	60.0%	< 0.001
Study location
Caucasia	6	1.17 (1.07–1.27)	0.692	0.0%	< 0.001
Asia	7	1.05 (1.01–1.09)	0.986	0.0%	< 0.001
Study design
Case–control	7	1.05 (1.01–1.09)	0.986	0.0%	< 0.001
Cohort	6	1.17 (1.07–1.27)	0.692	0.0%	< 0.001
Study quality
Score ≥ 7	7	1.16 (1.06–1.25)	0.669	0.0%	< 0.001
Score < 7	6	0.84 (0.65–1.03)	0.970	0.0%	0.316
Pancreas cancer	5	1.15 (1.05–1.19)	0.498	0.0%	< 0.001
Lung cancer	2	1.66 (1.34–1.97)	0.660	0.0%	< 0.001
Prostate cancer	2	1.14 (1.03–1.25)	0.481	0.0%	< 0.001
Bladder cancer	2	1.23 (1.12–1.35)	0.596	0.0%	< 0.001
Hematopoietic cancer	9	1.07 (1.02–1.13)	0.443	0.0%	< 0.001

### Tooth loss and esophageal cancer

**Figure 3:**
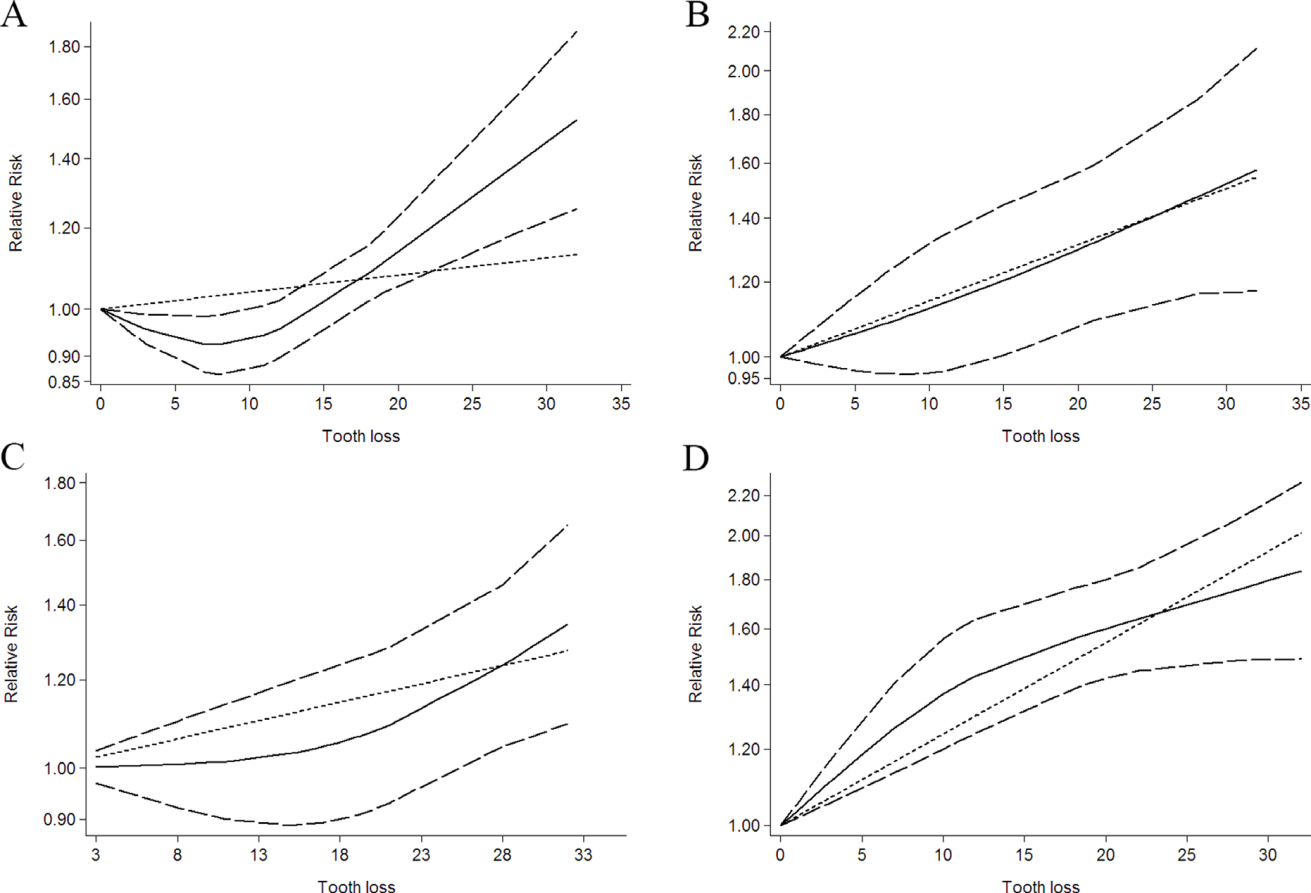
Dose-response relationship between tooth loss in relation to risk of cancer (**A**) Colorectal cancer. (**B**) Esophageal cancer. (**C**) Gastric cancer. (**D**) Head and neck cancer.

Ten independent reports from nine studies investigated the association between tooth loss and esophageal cancer [6–13, 24]. Compared with the lowest tooth loss, tooth loss is significantly associated with a higher risk of esophageal cancer (RR:1.18; 95% CI, 1.04–1.31; *P* < 0.001) (Table 2). Furthermore, tooth loss is significantly associated with esophageal cancer risk in Caucasia (RR:1.12; 95% CI, 1.02–1.21; *P* < .001) (Table 2) and Asia (RR:1.26; 95% CI, 1.08–1.44; *P* < .001) (Table 2). Additionally, a dose-response analysis revealed that each 10 tooth loss was associated with a 14% incremental in esophageal cancer risk (RR: 1.14; 95% CI, 1.05–1.25) (Figure 3). A cubic spline model revealed an positive non-linear correlation between tooth loss and esophageal cancer (*P* < 0.001 for non-linearity; Figure 3).

### Tooth loss and gastric cancer

Nine independent reports from five studies investigated the association between tooth loss and gastric cancer [6, 7, 12, 13, 15]. The results of tooth loss and gastric cancer risk are shown in Table 2. Compared with lowest the tooth loss, tooth loss is significantly associated with a higher risk of gastric cancer (RR: 1.09; 95% CI, 1.03–1.16; *P* < 0.001) (Table 2). Furthermore, tooth loss is significantly associated with gastric cancer risk in Caucasia (RR:1.25; 95% CI, 1.11–1.36; *P* < 0.001) (Table 2) and Asia (RR:1.04; 95% CI, 1.01–1.09; *P* < 0.001) (Table 2). Additionally, a dose-response analysis revealed that each 10 tooth loss was associated with a 9% incremental in gastric cancer risk (RR: 1.09; 95% CI, 1.01–1.18; *P* < 0.001) (Figure 3). A cubic spline model revealed an positive non-linear correlation between tooth loss and gastric cancer (*P* < 0.001 for non-linearity; Figure 3).

### Tooth loss and head and neck cancer

Nineteen independent reports from eleven studies investigated the association between tooth loss and head and neck cancer [11–13, 16–20, 23, 25, 26]. Compared with the lowest tooth loss, tooth loss is significantly associated with head and neck cancer risk (RR:1.52; 95% CI, 1.14–1.90; *P* < 0.001) (Table 2). Furthermore, tooth loss is significantly associated with a higher risk of head and neck cancer in Caucasia (RR:1.15; 95% CI, 1.04–1.26; *P* < 0.001) (Table 2) and Asia (RR:1.82; 95% CI, 1.52–2.12; *P* < 0.001) (Table 2). Also, tooth loss is significantly associated with oral cancer (RR:1.80; 95% CI, 1.30–2.30; *P* < 0.001) (Table 2), Pharynx cancer (RR:1.14; 95% CI, 1.05–1.23; *P* < 0.001) (Table 2) and Larynx cancer (RR:1.08; 95% CI, 1.02–1.15; *P* < 0.001) (Table 2). Additionally, a dose-response analysis revealed that each 10 tooth loss was associated with a 31% incremental in head and neck cancer risk (RR: 1.31; 95% CI, 1.15–1.50; *P* < 0.001) (Figure 3). A cubic spline model revealed an positive non-linear correlation between tooth loss and head and neck cancer cancer (*P* < 0.001 for non-linearity; Figure 3).

### Tooth loss and colorectal cancer

Thirteen independent reports from four studies investigated the association between tooth loss and colorectal cancer [12, 13, 28, 29]. Compared with the lowest tooth loss, tooth loss is significantly associated with a higher risk of colorectal cancer (RR:1.07; 95% CI, 1.02–1.14; *P* < 0.001) (Table 2). Furthermore, tooth loss is significantly associated with colorectal cancer risk in Caucasia (RR:1.17; 95% CI, 1.07–1.27; *P* < 0.001) (Table 2) and Asia (RR:1.05; 95% CI, 1.01–1.09; *P* < 0.001) (Table 2). Also, tooth loss is significantly associated with a higher risk of colon cancer (RR: 1.09; 95% CI, 1.02–1.17; *P* < 0.001) (Table 2) and rectal cancer (RR:1.08; 95% CI, 1.01–1.17; *P* < 0.001) (Table 2). Additionally, a dose-response analysis revealed that each 10 tooth loss was associated with a 4% incremental in colorectal cancer risk (RR:1.04; 95% CI, 1.01–1.08; *P* < 0.001) (Figure 3). A cubic spline model revealed an positive non-linear correlation between tooth loss and colorectal cancer (*P* < 0.001 for non-linearity; Figure 3).

### Tooth loss and pancreas cancer

**Figure 4:**
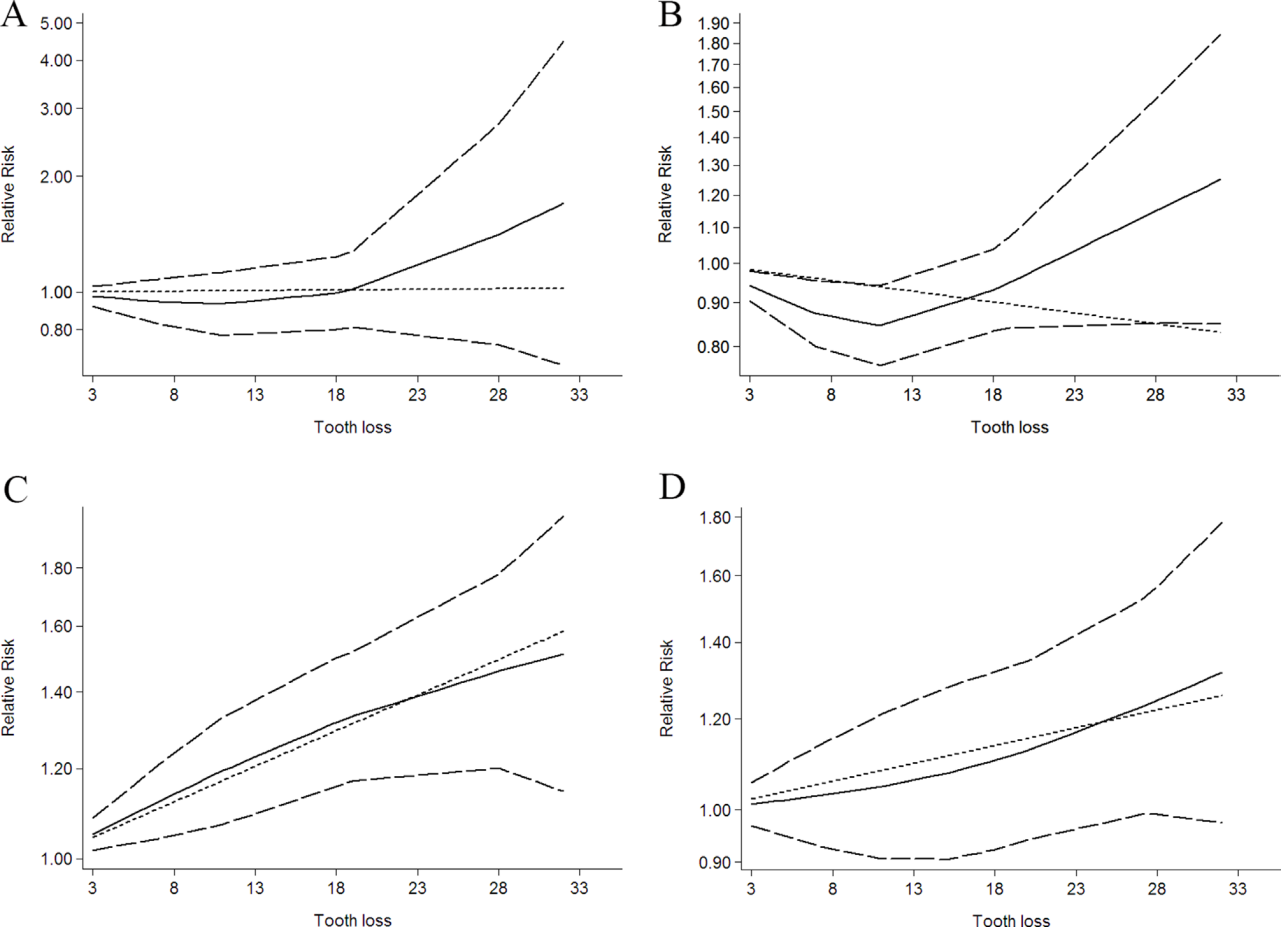
Dose-response relationship between tooth loss in relation to risk of cancer (**A**) Bladder cancer. (**B**) Hematopoietic cancer. (**C**) Lung cancer. (**D**) Pancreas cancer.

Five independent reports from five studies investigated the association between tooth loss and pancreas cancer [12–14, 21, 30]. Compared with the lowest tooth loss, tooth loss is significantly associated with a higher risk of pancreas cancer (RR:1.15; 95% CI, 1.05–1.19; *P* < 0.001) (Table 2). Additionally, a dose-response analysis revealed that each 10 tooth loss was associated with a 7% incremental in pancreas cancer risk (RR:1.07; 95% CI, 1.01–1.15; *P* < 0.001) (Figure 4). A cubic spline model revealed an positive non-linear correlation between tooth loss and pancreas cancer (*P* < 0.001 for non-linearity; Figure 4).

### Tooth loss and lung cancer

Five independent reports from two studies investigated the association between tooth loss and lung cancer [12, 13]. Compared with lowest tooth loss, tooth loss is significantly associated with a higher risk of lung cancer (RR: 1.66; 95% CI, 1.34-1.97; *P* < 0.001) (Table 2). Additionally, a dose-response analysis revealed that each 10 tooth loss was associated with a 19% incremental in lung cancer risk (RR:1.19; 95% CI, 1.04–1.35; *P* < 0.001) (Figure 4). A cubic spline model revealed an positive non-linear correlation between tooth loss and lung cancer (*P* < 0.001 for non-linearity; Figure 4).

### Tooth loss and bladder cancer

Two independent reports from two studies investigated the association between tooth loss and bladder cancer [12, 13]. Compared with lowest tooth loss, tooth loss is significantly associated with a higher risk of bladder cancer (RR: 1.23; 95% CI, 1.12–1.35; *P* < 0.001) (Table 2). Additionally, a dose-response analysis revealed that each 10 tooth loss was associated with a 2% incremental in bladder cancer risk (RR: 1.02; 95% CI, 1.01–1.03; *P <* 0.001) (Figure 4). A cubic spline model revealed an positive non-linear correlation between tooth loss and bladder cancer (*P* < 0.001 for non-linearity; Figure 4).

### Tooth loss and hematopoietic cancer

Two independent reports from two studies investigated the association between tooth loss and hematopoietic cancer [12, 13]. Compared with the lowest tooth loss, tooth loss is significantly associated with a higher risk of hematopoietic cancer (RR: 1.07; 95% CI, 1.02–1.13; *P* < 0.001) (Table 2). Additionally, a dose-response analysis revealed that each 10 tooth loss was associated with a 3% incremental in hematopoietic cancer risk (RR: 1.03; 95% CI, 1.01–1.07; *P* < 0.001) (Figure 4). A cubic spline model revealed an positive non-linear correlation between tooth loss and hematopoietic cancer (*P* < 0.001 for non-linearity; Figure 4).

### Subgroup analyses

Subgroup analysis was performed to check the stability of the primary outcome. Subgroup meta-analyses in study design and study quality showed consistent findings (Table 2).

### Sensitivity analysis

Sensitivity analysis was conducted to assess the stability of the results. The results show the results were stable in Supplementary Figure 1.

### Publication bias

Each studies in this meta-analysis were performed to evaluate the publication bias by both Begg’s funnel plot and Egger’s test. *P* > 0.05 was considered no publication bias. The results show no obvious evidence of publication bias was found in the associations between tooth loss and cancer risk (Supplementary Table 2).

## DISCUSSION

Cancer affects millions of people in developed and developing countries that is now a public health crisis. Despite the decline in the mortality rate of developed countries, cancer is still the main cause of death and has caused serious social and economic distress on a global scale over the past few decades. In low and middle-income countries, the incidence of cancer has risen sharply [2]. To date, there are a few identified risk factors for acute pancreatitis, including smoking, infection, occupational exposure, environmental pollution, unreasonable diet and genetic factors. Previous studies indicated that tooth loss may be a risk factors in cancer, but presented controversial results.

In the current meta-analysis was based on 25 case-control or cohort study, with more than 1.3 million participants and 32925 cases from eleven countries. Thus, this meta analysis provides the most up-to-date epidemiological evidence supporting tooth loss is harmful for cancer. A dose-response analysis revealed that a per 10 of tooth loss increase was associated with a 9% increment of cancer risk, 14% increment of esophageal cancer risk, 9% increment of gastric cancer risk, 31% increment of head and neck cancer risk, 4% increment of colorectal cancer risk, 7% increment of pancreas cancer risk, 19% increment of lung cancer risk, 3% increment of prostate cancer risk, 2% increment of bladder cancer risk and 3% increment of hematopoietic cancer risk. Subgroup meta-analyses by various factors also showed consistent findings.

Several plausible pathways may reasonable for the relationship between tooth loss and cancer. The influence of chronic inflammation on cancer development is one possible pathway. Chronic systemic inflammation linked to periodontal disease [36, 37], which is a major cause of tooth loss in adults that can increase the risk of cancer by inhibiting apoptosis and stimulating tumor cell proliferation [38]. Secondly, the main cause of teeth loss is dental caries [39, 40], and carbohydrate intake is the dental caries cause. Carbohydrate intake was associated with increased risk cancer [41, 42]. Third, the progress of tooth damage destroys normal periodontal tissue, allowing oral microbial accumulation deep into oral tissue, thereby promoting its growth [43]. Thus, tooth loss and cancer seems to be closely related.

To our knowledge, this is the first study to identify and quantify the potential dose-response association between tooth loss and cancer risk in a large cohort of both men and women. Although, we performed this meta-analysis very carefully, however, some limitations must be considered in the current meta-analysis. First, different sex of population should be included in this meta-analysis to explore the impact of different sex of population on tooth loss and cancer risk. Second, we only select literature that written by English, which may have resulted in a language or cultural bias, other language should be chosen in the further. Third, in the subgroup analysis in cancer type, there might be insufficient statistical power to check an association. Fourth, Though some data were obtained from retrospective studies, most of retrospective studies only to identify the relationship between tooth loss and cancer risk, and we also add more study to identify and quantify the potential dose-response association between tooth loss and cancer risk.

In conclusion, our findings underscore the notion that tooth loss was associated with cancer risk increment. In the future, large-scale and population based association studies must be performed in the future to validate the risk identified in the current meta-analysis.

## SUPPLEMENTARY MATERIALS FIGURE AND TABLES




