# Supplementary material for: Tooth loss and cancer risk: a dose–response meta analysis of prospective cohort studies
Source: Oncotarget. 2017 Dec 16;9(19):15090–100. doi: 10.18632/oncotarget.23850 (PMC5871100; doi:10.18632/oncotarget.23850)
Supplement: Supplementary file 1 [file oncotarget-09-15090-s001.pdf]

Tooth loss and cancer risk: a dose–response meta analysis of prospective cohort studies

SUPPLEMENTARY MATERIALS

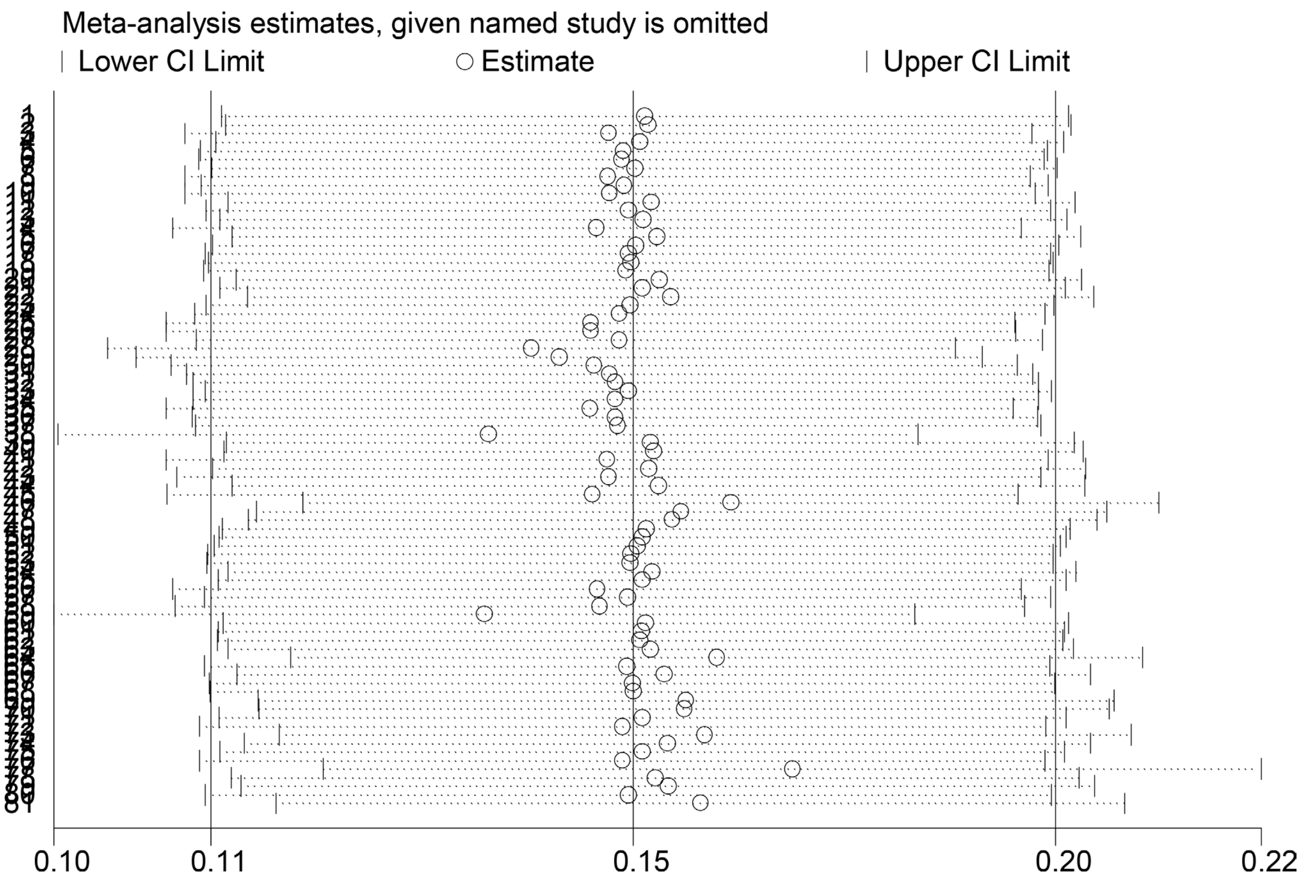

Supplementary Figure 1: Sensitivity analysis of the meta-analysis.

Supplementary Table 1: Outcomes and covariates of included studies of tooth loss in relation to risk of cancer. See Supplementary_Table_1

Supplementary Table 2: Publication bias analysis of the meta-analysis

	Test	t	95% CI	*P*
Cancer	Begg's test			0.128
	Egger's test	1.12	–0.29,1.03	0.267
Esophageal cancer	Begg's test			0.929
	Egger's test	1.27	–2.98,2.86	1.000
Gastric cancer	Begg's test			0.118
	Egger's test	0.89	–1.26,2.78	0.401
Head and neck cancer	Begg's test			0.327
	Egger's test	0.71	–1.54,3.10	0.487
Colorectal cancer	Begg's test			0.360
	Egger's test	-1.80	–2.82,0.27	0.099
Pancreas cancer	Begg's test			1.000
	Egger's test	-0.33	–7.94,6.45	0.764
Hematopoietic cancer	Begg's test			0.917
	Egger's test	0.25	–1.85,2.29	0.809
