# Supplementary material for: Tooth loss and cancer risk: a dose–response meta analysis of prospective cohort studies
Source: Oncotarget. 2017 Dec 16;9(19):15090–100. doi: 10.18632/oncotarget.23850 (PMC5871100; doi:10.18632/oncotarget.23850)
Supplement: Supplementary file 2 [file oncotarget-09-15090-s002.docx]

**Supplementary Table 1: Outcomes and covariates of included studies of tooth loss in relation to risk of cancer**

| **Author(year)** | **Endpoints** | **Data source** | **Category and relative risk (95% CI)** | **Covariates in fully adjusted model** |
| --- | --- | --- | --- | --- |
| Abnet et al(2005) | Esophageal cancer  Gastric cancer | Population-based | Esophageal cancer  <10 teeth lost, 1.0 (reference); >10-<31, 0.92 (0.46, 1.83); edentulous, 0.73 (0.35,1.55)  Gastric cardia adenocarcinoma  <10 teeth lost, 1.0 (reference); >10-<31, 0.86 (0.46, 1.60); edentulous, 0.93 (0.50,1.75)  Gastric non-cardia adenocarcinoma  <10 teeth lost, 1.0 (reference); >10-<31, 1.46 (0.97, 2.21); edentulous, 1.65 (1.09,2.49) | Adjusted for age at randomization and education |
| Abnet et al(2008) | Esophageal cancer | Population-based | <12 teeth lost, 1.0 (reference); >13-<18, 1.08 (0.59, 1.98);>19-<24, 1.46(0.81,2.65);  >25-<31, 1.02 (0.56, 1.87); edentulous, 1.79 (1.03,3.13) | Adjusted for age, sex, place of residence, ethnicity, alcohol drinking, use of tobacco, opium, or both, education in three categories, number of appliances, and fruit and vegetable intake |
| Dar et al(2013) | Esophageal cancer | Population-based | 0 teeth lost, 1.0 (reference); >1-<2, 1.29 (0.84, 1.98);>3-<5, 1.36(0.88,2.11);  >6, 1.08 (0.68, 1.69) | Adjusted for age, ethnicity, residence, education, wealth score, fruit and vegetable intake, bidi smoking, gutka chewing, alcohol consumption and cumulative use of hookah, cigarette, and nass |
| Guha et al(2007) | Esophageal cancer  Head and neck cancer | Hospital-based | Europe:  Esophageal cancer  <5 teeth lost, 1.0 (reference); >6-<15, 2.84 (1.26, 6.41);>16, 1.07 (0.41, 2.77)  Oral cavity cancer  <5 teeth lost, 1.0 (reference); >6-<15, 0.85 (0.45, 1.62);>16, 0.48 (0.22, 1.05)  Pharynx cancer  <5 teeth lost, 1.0 (reference); >6-<15, 1.04 (0.52, 2.09);>16, 0.81 (0.35, 1.87)  Larynx cancer  <5 teeth lost, 1.0 (reference); >6-<15, 1.00 (0.59, 1.70);>16, 0.56 (0.31, 1.01)  USA:  Esophageal cancer  <5 teeth lost, 1.0 (reference); >6-<15, 2.19 (1.04, 4.63);>16, 1.80 (0.80, 4.07)  Oral cavity cancer  <5 teeth lost, 1.0 (reference); >6-<15, 0.87 (0.56, 1.35);>16, 1.21 (0.77, 1.90)  Pharynx cancer  <5 teeth lost, 1.0 (reference); >6-<15, 1.45 (1.03, 2.05);>16, 1.29 (0.90, 1.86)  Larynx cancer  <5 teeth lost, 1.0 (reference); >6-<15, 1.67 (1.16, 2.42);>16, 1.69 (1.14, 2.51) | Adjusted for age, sex, country, education, tobacco pack-years, cumulative alcohol consumption, and all other oral health variables |
| Hiraki et al(2008) | Head and neck  Esophagus  Stomach  Colon  Liver  Pancreas  Lung  Breast  Uterus  Ovary  Prostate  Bladder  Thyroid  Lymphoma |  | Head and neck  <12 teeth lost, 1.0 (reference); >13-<24, 1.17 (0.88, 1.59);>19-<24, 1.31(0.88,1.93);  edentulous, 1.68 (0.88, 1.93)  Esophagus  <12 teeth lost, 1.0 (reference); >13-<24, 1.03 (0.72, 1.48);>19-<24, 1.93(1.24,3.01);  edentulous, 2.36 (1.17,4.75)  Stomach  <12 teeth lost, 1.0 (reference); >13-<24, 1.08 (0.87,1.35); >19-<24, 1.14 (0.85,1.52);  edentulous, 0.90 (0.58,1.41)  Colon  <12 teeth lost, 1.0 (reference); >13-<24, 1.22 (0.97,1.52); >19-<24, 1.11 (0.82,1.50);  edentulous, 0.92 (0.56,1.51)  Liver  <12 teeth lost, 1.0 (reference); >13-<24, 1.74 (1.04,2.89); >19-<24, 1.64 (0.90,2.98);  edentulous, 1.35 (0.51,3.58)  Pancreas  <12 teeth lost, 1.0 (reference); >13-<24, 1.33 (0.86,2.07); >19-<24, 0.60 (0.32,1.14);  edentulous, 1.33 (0.57,3.10)  Lung  <12 teeth lost, 1.0 (reference); >13-<24,1.02 (0.83,1.24); >19-<24, 1.22 (0.94,1.57);  edentulous, 1.54 (1.05,2.27)  Breast  <12 teeth lost, 1.0 (reference); >13-<24,0.89 (0.72,1.11) ; >19-<24, 0.95 (0.68,1.33);  edentulous, 0.79 (0.37,1.63)  Uterus  <12 teeth lost, 1.0 (reference); >13-<24,1.12 (0.81,1.54); >19-<24, 0.71 (0.45,1.13);  edentulous, 0.90 (0.43,1.88)  Ovary  <12 teeth lost, 1.0 (reference); >13-<24,0.95 (0.51,1.76); >19-<24, 0.92 (0.40,2.09);  edentulous, 0.18 (0.02,1.55)  Prostate  <12 teeth lost, 1.0 (reference); >13-<24,0.86 (0.51,1.47); >19-<24, 0.57 (0.29,1.12);  edentulous, 0.49 (0.19,1.26)  Bladder  <12 teeth lost, 1.0 (reference); >13-<24,0.80 (0.37,1.76); >19-<24, 1.16 (0.46,2.91);  edentulous, 2.85 (0.57,14.22)  Thyroid  <12 teeth lost, 1.0 (reference); >13-<24,1.05 (0.59,1.84); >19-<24, 2.15 (1.05,4.40);  edentulous, 1.27 (0.38,4.25)  Lymphoma  <12 teeth lost, 1.0 (reference); >13-<24,1.44 (0.94,2.21); >19-<24,1.34 (0.76,2.35);  edentulous, 1.17 (0.4,3.44) | Adjusted for age, sex, smoking and drinking status (never, former, current), vegetable and fruit intake, BMI, and regular exercise |
| Michaud et al (2008) | Total  Lung  Oropharyngeal  Esophageal(131)  Stomach(106)  Pancreatic(253)  Colorectal(1043)  Kidney(271)  Lung(678)  Bladder(5413)  Prostate(541)  Hematopoietic(934)  Brain(132)  Skin Melanoma(698)  Non-Hodgkin lymphoma(524)  Leukemia(250)  Multiple myeloma(141) |  | Total cancer  <7 teeth lost, 1.0 (reference); >8-<15,0.95 (0.88,1.02); >16-<32,1.09 (0.99,1.20)  Lung  <7 teeth lost, 1.0 (reference); >8-<15,1.34  (1.10,1.63); >16-<32,1.70 (1.37,2.11)  Oropharyngeal  <7 teeth lost, 1.0 (reference); >8-<15,1.18  (0.69,2.01); >16-<32,1.60 (0.84,3.04)  Esophageal  <7 teeth lost, 1.0 (reference); >8-<15,0.86  (0.51,1.43); >16-<32,1.34 (0.78,2.30)  Stomach  <7 teeth lost, 1.0 (reference); >8-<15,1.11  (0.65,1.88); >16-<32,1.10 (0.56,2.16)  Pancreas  <7 teeth lost, 1.0 (reference); >8-<15,1.08  (0.77,1.52); >16-<32,0.91 (0.56,1.47)  Colorectal  <7 teeth lost, 1.0 (reference); >8-<15,0.93  (0.78,1.12); >16-<32,1.10 (0.87,1.37)  Kidney  <7 teeth lost, 1.0 (reference); >8-<15,0.98  (0.77,1.24); >16-<32,1.00 (0.74,1.35)  Prostate  <7 teeth lost, 1.0 (reference); >8-<15,1.02  (0.81,1.28); >16-<32,0.70 (0.50,0.97)  Hematopoietic  <7 teeth lost, 1.0 (reference); >8-<15,0.93  (0.76,1.12); >16-<32,0.96 (0.74,1.24)  Brain  <7 teeth lost, 1.0 (reference); >8-<15,0.99  (0.58,1.70); >16-<32,1.31(0.66,2.59)  Skin Melanoma  <7 teeth lost, 1.0 (reference); >8-<15,0.75  (0.58,0.96); >16-<32,0.62(0.41,0.93)  Non-Hodgkin lymphoma  <7 teeth lost, 1.0 (reference); >8-<15,0.76  (0.58,1.00); >16-<32,0.80(0.56,1.16)  Leukemia  <7 teeth lost, 1.0 (reference); >8-<15,1.05  (0.73,1.50); >16-<32,1.02(0.62,1.70)  Multiple myeloma  <7 teeth lost, 1.0 (reference); >8-<15,1.28  (0.81,2.01); >16-<32,1.42(0.80,2.53) | Adjusted for age , race, physical activity, history of diabetes, alcohol, body mass index, geographic location, height , calcium intake (quintiles), total caloric intake (quintiles), red meat intake (quintiles), fruit and vegetable intake (quintiles), vitamin D score, smoking history (never, past quit ≤10 yrs, past quit >10 yrs, current 1–14 cig/d, 15–24 cig/d, 25+ cig/d) and pack-years (continuous) |
| Michaud et al (2007) |  |  | <7 teeth lost, 1.0 (reference); >8-<15,1.00  (0.67,1.48); >16-<32,1.02(0.61,1.71) | Adjusted for age, smoking history, profession, race, geographic location), physical activity, history of diabetes, body mass index, height, history of cholecystectomy, nonsteroidal anti-inflammatory drug use, multivitamin use, and in addition, for periodontal and tooth loss models, baseline teeth number, dietary intakes of fruits and vegetables, vitamin D, calcium, sucrose, and total calories |
| Shakeri et al(2013) | Gastric adenocarcinoma |  | Gastric adenocarcinoma  <12 teeth lost, 1.0 (reference); >13-<18, 0.5 (0.2, 1.1);>19-<24, 0.9(0.4,1.7); >25-<31, 1.6 (0.8, 3.2); edentulous, 1.4(0.6,3.0)  Gastric cardia adenocarcinoma  <12 teeth lost, 1.0 (reference); >13-<18, 0.6 (0.2, 1.9);>19-<24, 1.6(0.6,4.3); >25-<31, 3.5 (1.2, 9.7); edentulous, 1.4 (0.4,4.5)  Gastric non-cardia adenocarcinoma  <12 teeth lost, 1.0 (reference); >13-<18, 0.3 (0.1, 1.2);>19-<24, 0.4(0.1,1.3); >25-<31, 1.7 (0.5, 5.6); edentulous, 2.1 (0.6,6.9) | Adjusted for age, ethnicity, education fruit and vegetable use, socioeconomic status, ever opium or tobacco use, and denture use |
| Balaram et al(2002) | oral cavity |  | Men  <5 teeth lost, 1.0 (reference); >5,3.89  (2.46,6.17)  Women  <5 teeth lost, 1.0 (reference); >5,7.61  (3.89,14.88) | Adjusted for age, center, education and (men only) smoking and drinking habits |
| Bundgaard et al(1995) | oral cavity |  | <17 teeth lost, 1.0 (reference); >18-<27, 1.9 (1.1, 3.4);>28-<32, 2.4(1.3,4.1) | Unadjusted |
| Garrote et al(2001) | oral cavity |  | <5 teeth lost, 1.0 (reference); >6-<15, 1.82 (0.76, 4.35);>16-<32, 2.74(1.23,6.12) | Adjusted for gender, age, area of residence, education, smoking and drinking habits |
| Lissowska et al(2003) | oral cavity |  | <5 teeth lost, 1.0 (reference); >6-<15, 7.00  (1.68, 29.11);>16-<32, 9.85(2.26,42.84) | Adjusted for age,gender, residence, smoking and drinking habits. |
| Talamini et al(2000) | oral cavity |  | <5 teeth lost, 1.0 (reference); >6-<15, 1.1  (0.5, 2.6);>16-<32, 1.4(0.6,3.1) | Adjusted for gender, age, fruit and vegetable intake and smoking and drinking habits |
| Stolzenberg-Solomon et al(2003) | pancreatic cancer |  | <10 teeth lost, 1.0 (reference); >11-<31, 1.23  (0.82, 1.85); edentulous, 1.63 (1.09,2.46) | Adjusted for age, number of years of smoking, education, urban living, and height |
| Bertrand et al(2017) | NonHodgkin lymphoma  Chronic lymphocytic leukemia/small lymphocytic lymphomas  Diffuse large B-cell lymphomas  Follicular lymphomas |  | NonHodgkin lymphoma  <7 teeth lost, 1.0 (reference); >8-<15, 0.75  (0.60, 0.94); >16-<32,0.80(0.59,1.09)  Chronic lymphocytic leukemia/small lymphocytic lymphomas  <7 teeth lost, 1.0 (reference); >8-<15, 0.50  (0.32, 0.79); >16-<32,0.57(0.31,1.05)  Diffuse large B-cell lymphomas  <7 teeth lost, 1.0 (reference); >8-<15,1.13  (0.59, 2.16); >16-<32,0.39(0.09,1.69)  Follicular lymphomas  <7 teeth lost, 1.0 (reference); >8-<15, 0.79  (0.37, 1.68); >16-<32, 1.77(0.77,4.03) | Adjusted for age, race, diabetes history, body mass index, geographic region, current smoking status (never, past, current), and NSAID use at baseline |
| Chen et al(2016) | oral cavity |  | Illiterate  0 teeth lost, 1.0 (reference); <5,2.53  (0.99, 6.48); >5, 2.84(1.10,7.34)  Primary-middle school  0 teeth lost, 1.0 (reference); <5, 1.55  (0.87, 2.77); >5, 2.65(1.48,4.73)  High school and above  0 teeth lost, 1.0 (reference); <5, 1.06  (0.48, 2.34); >5, 2.23(1.01,4.95) | Adjustment for age, marital status, residence, family history of cancer, passive smoking, exposure to cooking oil fumes, and diet containing vegetables and fruit. |
| Chen et al(2016) | Esophageal |  | 0 teeth lost, 1.0 (reference); 1><6, 1.12  (0.81, 1.56); >7, 1.39(0.96, 2.00) | Adjusted for age, sex, education, marital status, tobacco smoking,alcohol drinking, tea drinking, family history of ESCC, daily consumption of pickled vegetables, daily consumption of fresh fruits, and wealth score. |
| Zuo et al(2014) | Oral cancer |  | <5 teeth lost, 1.0 (reference); 6><15, 1.06  (0.53, 2.13); >16, 3.64(1.15, 11.53) | Adjusted for age at diagnosis, gender, smoking, alcohol use, body mass index, and history of diabetes mellitus |
| Liu et al(2016) | nasopharyngeal carcinoma |  | <5 teeth lost, 1.0 (reference); 1><3, 0.97  (0.84, 1.11);4><13, 0.99 (0.83, 1.18); >14, 1.00(0.75, 1.32) | Adjusted for sex, age, residential area, education level, current housing type, current occupation, first-degree family history of nasopharyngeal carcinoma, cigarette smoking, tea drinking, and salt-preserved fish consumption in 2000-  2002. |
| Momen-Heravi et al(2017) | colorectal cancer |  | Colorectal cancer  <7 teeth lost, 1.0 (reference); 8><15, 0.94  (0.80, 1.09); >16, 1.20(1.04, 1.39)  Colon cancer  <7 teeth lost, 1.0 (reference); 8><15, 0.88  (0.74, 1.04); >16, 1.14(0.97, 1.35)  Proximal colon cancer  <7 teeth lost, 1.0 (reference); 8><15, 0.89  (0.72, 1.10); >16, 1.23(1.01, 1.51)  Distal colon cancer  <7 teeth lost, 1.0 (reference); 8><15, 0.86  (0.64, 1.16); >16, 1.03(0.76, 1.38)  Rectal cancer  <7 teeth lost, 1.0 (reference); 8><15, 1.20  (0.88, 1.67); >16, 1.48(1.07, 2.05) | Adjusted for age, race, smoking before age 30, history of colorectal cancer in a parent or sibling , history of sigmoidoscopy/colonoscopy, current physical activity, regular aspirin use,  multivitamin use , type 2 diabetes, alcohol consumption, adult BMI, energy-adjusted intake of total calcium, vitamin D, folate, red meat and  processed meat and postmenopausal hormone use. |
| Ren et al(2016) | colorectal cancer |  | SWHS/SMHS study  Colorectal cancer  0 teeth lost, 1.0 (reference); 1><5, 0.88  (0.70, 1.11);6><10, 0.92 (0.69, 1.23); >10, 0.81(0.63, 1.06)  Colon cancer  0 teeth lost, 1.0 (reference); 1><5, 0.88  (0.64, 1.20);6><10, 0.95 (0.65, 1.38); >10, 0.83(0.58, 1.18)  Rectum cancer  0 teeth lost, 1.0 (reference); 1><5, 0.89  (0.63, 1.27);6><10, 0.85 (0.54, 1.36); >10, 0.80(0.53, 1.21)  SCCS study  Colorectal cancer  0 teeth lost, 1.0 (reference); 1><5, 1.14  (0.72, 1.79);6><10, 0.87 (0.52, 1.43); >10, 1.00(0.63, 1.58)  Colon cancer  0 teeth lost, 1.0 (reference); 1><5, 1.32  (0.75, 2.30);6><10, 0.98 (0.54, 1.80); >10, 0.99(0.56, 1.73)  Rectum cancer  0 teeth lost, 1.0 (reference); 1><5, 0.67  (0.27, 1.67);6><10, 0.53 (0.19, 1.45); >10, 0.98(0.41, 2.34) | In SMHS/SWHS, models were adjusted for income, education, BMI, exercise, smoking (never, former, and current), and red meat and fruit consumption  In SCCS, models were adjusted for occupation, income, education, and fruit and sweet beverage consumption |
| Huang et al(2016) | pancreatic cancer(126) |  | <11 teeth lost, 1.0 (reference); 12><21, 1.2  (0.7, 2.0); >22, 1.3(0.7,2.3) | Models were adjusted for age, sex and attained calendar period in 10-year-intervals, tobacco use , alcohol consumption, and area of residence |
